# Evaluation of Control Strategies for Porcine Reproductive and Respiratory Syndrome (PRRS) in Swine Breeding Herds Using a Discrete Event Agent-Based Model

**DOI:** 10.1371/journal.pone.0166596

**Published:** 2016-11-22

**Authors:** Andréia Gonçalves Arruda, Robert Friendship, Jane Carpenter, Amy Greer, Zvonimir Poljak

**Affiliations:** 1 Department of Population Medicine, University of Guelph, Guelph, Ontario, N1G 2W1, Canada; 2 Woodstock, Ontario, N4S 6N8, Canada; Atlantic Veterinary College, CANADA

## Abstract

The objective of this study was to develop a discrete event agent-based stochastic model to explore the likelihood of the occurrence of porcine reproductive and respiratory syndrome (**PRRS**) outbreaks in swine herds with different PRRS control measures in place. The control measures evaluated included vaccination with a modified-live attenuated vaccine and live-virus inoculation of gilts, and both were compared to a baseline scenario where no control measures were in place. A typical North American 1,000-sow farrow-to-wean swine herd was used as a model, with production and disease parameters estimated from the literature and expert opinion. The model constructed herein was not only able to capture individual animal heterogeneity in immunity to and shedding of the PRRS virus, but also the dynamic animal flow and contact structure typical in such herds under field conditions. The model outcomes included maximum number of females infected per simulation, and time at which that happened and the incidence of infected weaned piglets during the first year of challenge-virus introduction. Results showed that the baseline scenario produced a larger percentage of simulations resulting in outbreaks compared to the control scenarios, and interestingly some of the outbreaks occurred over long periods after virus introduction. The live-virus inoculation scenario showed promising results, with fewer simulations resulting in outbreaks than the other scenarios, but the negative impacts of maintaining a PRRS-positive population should be considered. Finally, under the assumptions of the current model, neither of the control strategies prevented the infection from spreading to the piglet population, which highlights the importance of maintaining internal biosecurity practices at the farrowing room level.

## Introduction

Despite the progress in porcine reproductive and respiratory syndrome (**PRRS**) research in the last 25 years, this remains the most significant endemic swine disease in North America, and elimination of the causative agent from distinct populations is complicated. Major knowledge gaps still exist with regard to this important disease [1], not only at the individual animal level but also at the herd level. Control and elimination methods have been developed in the last few years [2], and these are mainly focused on the use of live-virus inoculation (**LVI**) or modified-live vaccination (**MLV**) when depopulation is not an option. It is known that none of the commercially available MLV is labeled to prevent infection, but they reduce the duration of shedding and clinical signs of PRRS [3]. One of the major impediments to PRRS control and possible eradication is the persistence of PRRS infection in individual pigs [4]. Even though control and elimination methods have been previously described and are commonly used in field settings [5], results on the comparative effectiveness of different methods are scarce [3]. An important reason for the lack of head-to-head studies is that it would be unethical to compare PRRS control methods using intentional virus challenge in a field trial. Furthermore, it is extremely challenging to reach a counterfactual state between swine herds in order to obtain valid comparisons and conclusions.

Mathematical modelling is becoming more common in the veterinary sciences, and is an attractive alternative for situations where traditional epidemiological studies are unfeasible under field conditions. It allows for the assessment of potential consequences of disease introduction and for the testing of control strategies on simulated outbreaks. Mathematical models can be deterministic or stochastic. One of the important differences between them is that deterministic models describe what happens on average to a particular population, while stochastic models embrace the variability between individuals in such a population and incorporate chance, allowing for the description of outliers [6]. Stochastic compartmental models have been previously developed for PRRS [7–9]. Nodelijk et al. (2000) used stochastic “SIR” (susceptible-infected-recovered, referring to the compartments in which the population is stratified) models to explore PRRS virus (PRRSV) transmission and time-to-extinction using a 115-sow breeding herd as a case study. In addition, Evans et al. (2010) investigated within-herd transmission of PRRS focusing on infection persistence and fade-out of infections, while accounting for different contact patterns among ages. Both publications used European farrow-to-finish swine facilities as examples. More recently, Jeong et al. (2014) developed models to evaluate the effectiveness of control strategies that included herd closure with or without gilt acclimation, and single or repeated mass immunization with a modified-live vaccine, using a typical Midwestern US farrow-to-wean herd as a case study.

Even though models such as the ones described above are able to describe dynamics of infection in populations, discrete event agent-based models in particular are especially useful for describing complex diseases such as PRRS for three main reasons. The agent-based approach allows for consideration of biological variability between individuals, local interactions, life cycles, and behavior adapting to the individual’s changing internal and external environment [10]. These models are, in nature, stochastic [11]. As such, disease parameters such as duration of immunity can be drawn in an individual basis from relevant distributions in order to better reflect the variability within the population of interest. Discrete events can also be incorporated into these models, and spatial location, for example, can be explicitly taken into account. Connectedness between agents can also be modeled and updated as animals move through different locations and stages of production. The use of agent-based approaches combined with explicit discrete event modeling has never been previously explored for PRRS to the knowledge of the authors.

The main goal of the present study was to develop a discrete event agent-based model to evaluate control strategies for PRRS in the case of a field virus re-introduction, using a typical North American farrow-to-wean swine herd as a case study. Control strategies explored herein included vaccination of gilts with a modified live-attenuated vaccine, and live-virus inoculation of gilts. Secondary objectives were to explore how the number of infected animals introduced into the herd, and how immunization efficacy would change the likelihood of outbreaks occurring on a swine farm. Both of these objectives are aligned with a frequently asked question about the likelihood of introducing a novel strain and major outbreak in a swine herd that is purposively kept in the state of high herd immunity against PRRSV.

## Materials and Methods

### Purpose

A discrete event agent-based model was developed using the software Anylogic^®^ version 7.1.2 (XJ Technologies, St Petersburg, Russia). The main purpose of the model was to evaluate different PRRS control strategies for previously infected PRRS sites using a 1,000-sow farrow-to-wean North American herd as an example. The model description outlined in the next paragraphs follows general reporting guidelines from Grimm et al. (2006).

### State variables and scales

The hierarchical levels considered in this model were individuals (two types of so-called ‘agents’: female pigs and their offspring; piglets) and the environment.

**Fig 1:**
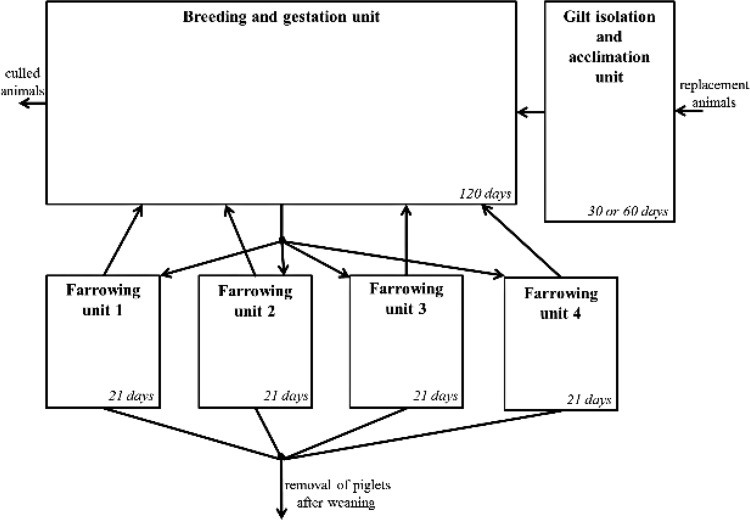
Immunological state of animals (female pigs and piglets) for the different control scenarios, and baseline. A. Baseline and live-virus inoculation scenario for female pigs; B. Modified-live vaccine scenario for female pigs and C. Immunological state for piglets (all scenarios); ^1^Duration followed a distribution, please refer to Table 1.

Individual adult female pigs were characterized by mutually exclusive PRRS immunological status: ‘susceptible’, ‘infected’, or ‘recovered’. In cases where the vaccination scenario was evaluated, female pigs could also be characterized as ‘vaccinated’ or ‘recovered by vaccination’. Immunity against both vaccine strain and field strain viruses was not considered to be life-long, but assumed to wane over time. Individual piglets were similarly characterized by mutually exclusive PRRS status: ‘susceptible’, ‘infected’, or ‘immune’. There was no waning of immunity for piglet agents. All possible PRRS immunological statuses for each control scenario are shown on Fig 1.

The environment was composed of six rooms, which were modeled as discrete locations where individuals would spend varying amounts of time. The first room was the isolation/ acclimation room, where all breeding female pigs arrived in the herd and spent their first days; the second room was the breeding unit, where female pigs were bred and gestated; and finally four farrowing rooms where female pigs gave birth to the piglet agents, and where those agents would spend all their time. The four farrowing rooms followed an “all-in, all-out” schedule, which means that a group of female pigs would move into one of these rooms and remain there for a fixed period of three weeks. Hence, only one of the four rooms per week would be receiving new female pigs, and available rooms would rotate on a weekly basis.

### Process overview and scheduling

The model proceeded in daily time steps, with a time frame of 1,730 days. A total of 1,000 days was allocated for an initial model run (model ran for the first 1,000 days to assure a stable population before the introduction of infected animals), and the following 730-day period corresponded to the herd follow-up after challenge (virus introduction), a period of approximately two years.

**Fig 2:**
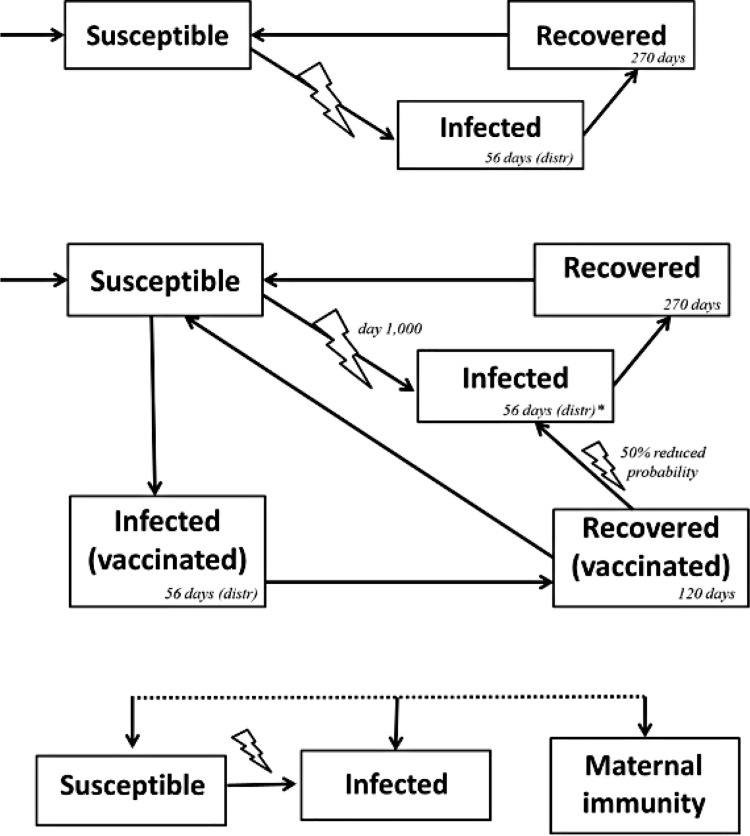
Schematic of the farrow-to-wean swine herd used as a model showing pig flow and spatial structure assumed in the developed discrete event agent-based stochastic model.

Replacement of the herd female pigs was considered in the model, with incoming gilts introduced into the herd once monthly (45 animals per time; replacement rate of 50% of the herd per year) through the isolation/ acclimation unit. Individuals entering the herd were assumed to be susceptible and remained in this physical space for a period of 30–60 days (depending on the control measure being evaluated), and proceeded to the breeding/ gestation unit, where they remained for a fixed period of 120 days. After this period, animals proceed to one of four farrowing rooms, according to the previously described sequential “on-off” room schedule, where they farrowed (gave birth to piglet agents). Piglet agents stayed exclusively in the farrowing room where they were born for a fixed period of 21 days, when they then left the herd. At this same point, female pigs returned to the breeding/ gestation unit and re-started their reproductive cycle. Female pigs were removed from the system (culling or death) at a constant rate that was the same as the replacement rate (50% per year) in order to keep a stable number of animals in the herd; and at all times removal was conducted from the breeding/ gestation unit. A schematic of pig flow and room locations is presented on Fig 2.

### Design concepts

#### Porcine reproductive and respiratory syndrome transmission dynamics

For the purpose of this manuscript, it is important to make the distinction between infection and disease; the first refers to the moment when the virus invades the host while the second refers to the development of clinical signs. Infectiousness, therefore, is directly linked to virus shedding and, therefore, transmissibility; while virulence is reflective of how likely the agent is to cause severe disease. In the current manuscript, infection was being modeled, and not disease. The rationale was that control measures were focused on reducing transmission and duration of persistence of the virus in a herd, and not on reduction of clinical signs. Stochasticity was incorporated not only in contact rate between animals, but also in the rate at which animals recover from disease (duration of infectiousness, drawn from a triangular distribution), and in the rate at which immunity wanes (duration of immunity, drawn from a Pert distribution). These parameters were selected to be stochastic because duration of PRRSV shedding and duration of immunity against infection with a field PRRSV strain are highly variable and uncertain.

In order to account for vertical transmission of PRRSV between sows and piglets, it was assumed that all susceptible animals would give birth to susceptible piglets, piglets born to immune sows were born with maternal immunity, and piglets born to infected sows were born infected [9]. Finally, piglets born to recovered vaccinated sows were assumed to be born immune, and piglets born to vaccinated sows that were still infectious were assumed to be born susceptible. As previously mentioned, waning of maternal immunity was not considered due to the fact that piglets only remained in the premises for a short period of time.

#### Environment and contact network

Interactions between agents were modeled based on agent’s location. Random probability of contact was assumed between animals within the same physical location. However, to account for the fact that there is variability in the number of contacts made per day amongst animals, contact rate (number of contacts per day) was a stochastic parameter following a triangular distribution (Table 1). Due to the fact that PRRSV transmission between rooms is realistic to assume (e.g. through movement of animals, people, airborne transmission, among others [12]), animals located in different physical locations had the possibility of contacting others and potentially spreading PRRS infection. This transmission probability was considered to be 10,000 times smaller compared to the transmission between animals housed in the same physical space, as previously assumed by Evans et al. (2010).

**Table 1:** Definition of parameters and values used for model simulations, and their references.

Parameter	Value (unit)	Reference
Contact rate	Triangular distribution, mode 5 contacts/ day, min 2 contacts/ day and 15 max contacts/ day	Expert opinion
Duration of infectiousness	Triangular distribution, mode 56 days, min 7 days and max 250 days	[7],[13], expert opinion
Duration of infectiousness for vaccinated animals	Triangular distribution, 30% reduction: mode 39 days, min 7 days and max 175 days	[14],[9], expert opinion
Duration of infectiousness of vaccine virus strain	Triangular distribution, mode 56 days, min 7 days and max 250 days	[4]
Duration of immunity	Pert distribution, mode 252 days, min 182 days and max 364 days	[8], expert opinion
Duration of immunity from the vaccine for vaccinated animals	120 days	Expert opinion, vaccine labels
Probability of infection with a field strain for naïve animals	40%	[15]
Probability of infection with a field strain for vaccinated animals	50% reduction of 40% = 20%	Expert opinion
Replacement Rate	50% per year	Expert opinion
Immunization efficacy	95%^a^	Expert opinion
Number of piglets weaned per susceptible/ immune/ vaccinated sows	12 piglets	[9]
Number of piglets weaned per infected sow	6 piglets	[9]
Probability of infection for vaccinated animals	Ten times reduction of 40% = 4%	[16]

^a^Sensitivity analysis conducted for the modified-virus vaccination scenario.

### Initialization

The evaluation of each disease control scenario began after the challenge-virus introduction (in a randomly selected environment of the site) on day 1,000 for each simulation. Before the infectious animal was introduced into the herd, the model ran for 1,000 days to assure demographic equilibrium. The time of initialization was the same for all scenarios, but immunological statuses amongst animals differed for the vaccination, live virus exposure and naïve herd scenarios, due to the nature of each control measure. For the vaccination scenario, a percentage of the population was partially immune, because a certain percentage (depending on the scenario) of animals entering in the herd were vaccinated with a heterologous virus strain, which offered some immunity against the field virus. For the virus exposure scenario, part of the population was completely immune, because a large proportion of animals entering the herd were exposed to a homologous virus strain, and for the naïve herd scenario, all animals within the population were susceptible.

### Inputs

Model inputs were obtained from the peer-reviewed literature on previous challenge studies, field observations and, when not available, obtained from discussions with experts in the area of swine production. The panel of experts included three people, all three were swine veterinarians, with one being primarily a field veterinarian and past coordinator of PRRS control projects, and the other two academicians with experience in PRRS research and field expertise. The information was elicited via a set of in person meetings and discussions carried out by the primary author of this publication. A list of inputs used in the model, as well as their references, is shown in Table 1.

### Model calibration and statistical analysis of outcomes

Since empirical information was unavailable, the model was calibrated to reproduce plausible values, and those were reviewed by a panel of three experts that included field veterinarians and researchers. Sensitivity analysis was considered an important component of model results, since it is recognized there are considerable knowledge gaps in PRRS epidemiology.

Data processing was conducted using Excel^®^ and Stata 13. Descriptive statistical analyses were conducted using Stata 13 and SAS version 9.3. These included graphical representation of the simulations in regards to number of infectious animals over time, histograms and boxplots representing the maximum number of animals infected (indication of presence of outbreaks), and histograms and boxplots representing the yearly incidence of infected weaned piglets (in log scale).

### Simulation experiments

Each scenario consisted of 1,000 replicates. Outcomes measured included the maximum number of females infected per simulation and time at which that happened, and the incidence of infected weaned piglets during the first year of challenge virus introduction. For boxplot graphs, the log 10 of the incidence rates was used. In total, ten scenarios were investigated, including changes in specific parameters for sensitivity analysis (Table 2). Further explanations of the three main scenarios are detailed in the following paragraphs.

**Table 2:** Description of porcine reproductive and respiratory syndrome control scenarios investigated in the developed discrete event agent-based stochastic model.

Scenario^a^	Immunization method	Duration of isolation/ acclimation	Immunization efficacy	Number of infected animals introduced in the herd
Baseline^b^_1	None	60 days	-	1
Baseline^b^_5	None	60 days	-	5
Baseline^b^_10	None	60 days	-	10
LVI^c^_60_95_a	LVI, I = distr^d^	60 days	95%	5
LVI^c^_60_95_b	LVI, I = 56d^e^	60 days	95%	5
LVI^c^_30_95	LVI	30 days	95%	5
MLV^f^_60_95	MLV	60 days	95%	5
MLV^f^_60_80	MLV	60 days	80%	5
MLV^f^_60_70	MLV	60 days	70%	5
MLV^f^_30_95	MLV	30 days	95%	5

^a^Each simulation consisted of 1,000 replicates

^b^No control scenarios in place

^c^Live-virus inoculation, assuming the field virus strain is known and can be isolated, and is homologous to future virus challenge

^d^Duration of infectiousness follows a triangular distribution, with a mode of 56 days, a minimum of 7 days and a maximum of 250 days

^e^Duration of infectiousness fixed at 56 days

^f^Modified-live attenuated vaccination.

#### Baseline scenario

As previously mentioned, the baseline scenario was developed to describe the dynamics of infection in a situation where no PRRS control measures were in place, reflecting a completely naïve (susceptible) population of pigs. Such scenario is likely to occur in newly established herds, or herds that had a PRRS virus introduction and conducted a herd closure and rollover strategy to eliminate the virus without complete depopulation. Herd closure and rollover strategy refers to cases where, after infection, the whole herd is closed for a period of time (at least 6 months there is no introduction of animals) and all animals are exposed to the virus at the same time (either via live-virus inoculation or vaccination) [2]. This procedure focus on ensuring that all animals mount an immune response and therefore transmission can be decreased [2]. After the closed period, replacement animals are introduced to the herd and when all the previously infected animals are replaced the process of herd “rollover” is complete. A total of one, five or ten infected animals were introduced into the herd, respectively, in separate simulations to allow for the evaluation of the secondary objective. Additional sensitivity analysis was conducted for this scenario to evaluate the impact of changing: (i) number of contacts, (ii) baseline probability of infection, and (iii) probability of infection between pigs located in different rooms on the cumulative incidence.

#### Modified live-virus vaccination (MLV) scenario

For the MLV scenario, it was assumed that animals receiving vaccination had a decreased probability of infection with live virus by half compared to naïve animals, and that vaccination reduced duration of infectiousness by 30% for cases where animals became infected ([14]; Table 1). Vaccination occurred as soon as animals entered the isolation/ acclimation room, and they received the vaccine only once. Sensitivity analysis was carried out using duration of acclimation of 30 and 60 days separately, and using vaccine efficacies of 95%, 80% and 70%. There was no discrimination between vaccine coverage or vaccine effectiveness for the purpose of this differentiation, therefore ‘immunization efficacy’ can be interpreted as a combination of both.

#### Live-virus inoculation (LVI) scenario

Similarly to the scenario described above, for the LVI scenario all incoming animals (replacement gilts) were exposed to the virus as soon as they entered the herd, with an immunization efficacy of 95% and administered once. For sensitivity analysis, two scenarios were simulated using duration of acclimation of 30 and 60 days separately, and both using duration of infectiousness following a triangular distribution; and one scenario was run using duration of acclimation of 60 days, and a fixed duration of infectiousness of 56 days. This last simulation was constructed because under field conditions, it is commonly assumed that animals will shed the virus for an approximate duration of 50–60 days. It is important to note that, for this scenario, even though a constant PRRS positive population is maintained in the herd at all times, the animals intentionally exposed via live-virus inoculation are not counted as part as the animals infected by introduction of challenge virus during collection of outcome measures. A complete list of production and disease parameters used in the model is shown in Table 1.

It is important to note that there was no herd closure for any of the scenarios at any point in time, and that the newly introduced challenge virus was considered to be homologous to the virus used in the LVI strategy, but heterologous to the virus used in the MLV strategy.

## Results

During a period of one year, across all scenarios, a mean minimum of 19,452 and a mean maximum of 25,911 piglets were produced for the 1,000 simulations. This is within the expected value considering a herd size varying between 900–1,200 sows, and the fact that a sow produces between 2 and 3 litters per year, with 6–10 piglets weaned per litter (expected value lies between a minimum of 10,800 and a maximum of 36,000).

### Baseline scenario

**Fig 3:**
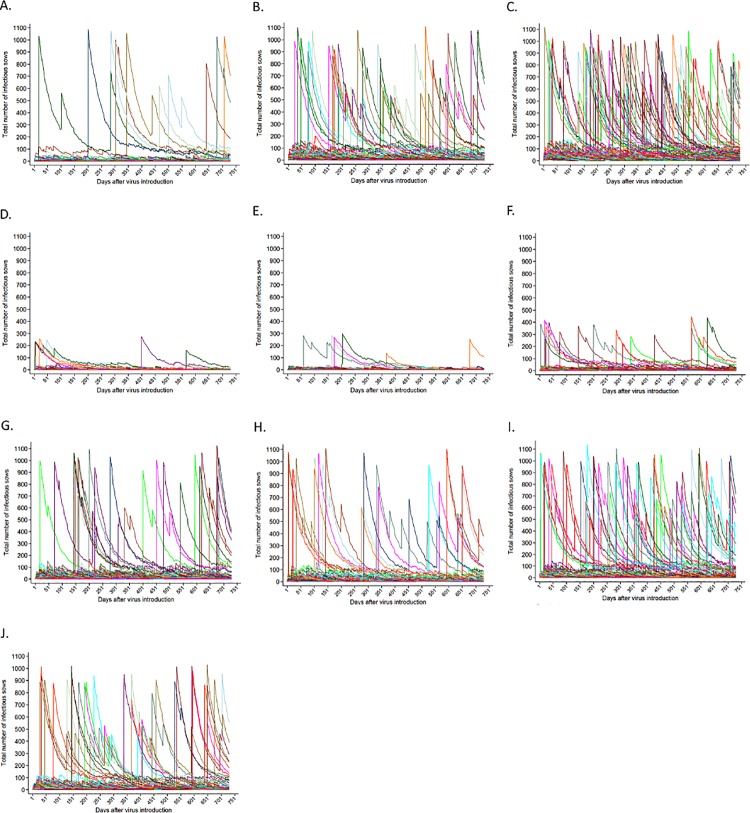
Porcine reproductive and respiratory syndrome dynamics as predicted by the model for different scenarios after virus introduction. **Figures contain 100 simulations only for easier visualization.** A. Baseline 1; B. Baseline 5; C. Baseline 10; D. Live-virus inoculation, 30d acclimation; E. Live-virus inoculation, 60d acclimation; F. Live-virus inoculation, 60d acclimation, 56d for duration of infectiousness; G. Modified-live vaccine scenario, 95% immunization efficacy and 60 days of duration of acclimation; H. Modified-live vaccine scenario, 80% immunization efficacy and 60 days of duration of acclimation; I. Modified-live vaccine scenario, 70% immunization efficacy and 60 days of duration of acclimation; J. Modified-live vaccine scenario, 95% immunization efficacy and 30 days of duration of acclimation. Please note that colors may be repeated for different outbreaks.

As expected, the baseline scenarios produced a greater percentage of simulations that resulted in outbreaks compared to the other scenarios. This percentage increased as the number of infected animals introduced into the herd increased (Fig 3A–3C). On average, the maximum number of female pigs infected at one-point-in-time (prevalence measure), considering a period of approximately two years, was 99.5 (SD: 273.6), 321.2 (SD: 424.1) and 489.4 (SD: 445.2) when one, five and ten infected females were introduced into a totally naïve herd, respectively (Table 3). It is important to note that this measure does not refer to the cumulative incidence, but the maximum number of infectious animals in the herd at a certain point in time. For all scenarios, but most evident for the scenario where one infected animal was introduced, the distribution was highly right skewed, with the 50^th^ percentile being 5, 75 and 143 female pigs, respectively (Table 3).

**Table 3:** Percentage of simulations binned according to the maximum numbers of infected females (at one point-in-time) for all scenarios evaluated in the developed discrete event agent-based stochastic model, considering a period of two years. E.g.: for baseline scenario with one infected female being introduced, 8% of the simulations produced a maximum number of infected female pigs of 800 or more.

	Maximum number of infected animals										
Scenario^a^	1–10	11–50	51–100	101–200	201–300	301–400	401–500	501–600	601–700	701–800	>800
Baseline_1	53.3	30.0	7.5	1.2	0	0	0	0	0	0	8.0
Baseline_5	3.0	25.3	36.4	8.2	0.1	0	0	0	0	0	27.1
Baseline_10	0.1	4.7	29.4	20.9	0.3	0.3	0	0	0	0.1	44.2
LVI_30_95	82.3	17.0	0.1	0	0.6	0	0	0	0	0	0
LVI_60_95_a	81.5	17.9	0	0.1	0.5	0	0	0	0	0	0
LVI_60_95_b	68.5	29.9	0.3	0	0.3	0.7	0.3	0	0	0	0
MLV_60_95	5.4	30.0	33.6	9.0	0.1	0.2	0	0	0	0	21.7
MLV_60_80	4.9	29.4	35.3	7.2	0.1	0.1	0	0	0	0	23.0
MLV_60_70	5.7	31.3	32.9	5.1	0.1	0	0	0	0	0	24.9
MLV_30_95	4.6	30.4	36.1	6.0	0.2	0	0	0	0	0.2	22.5

^a^For scenario definitions please refer to Table 2.

**Fig 4:**
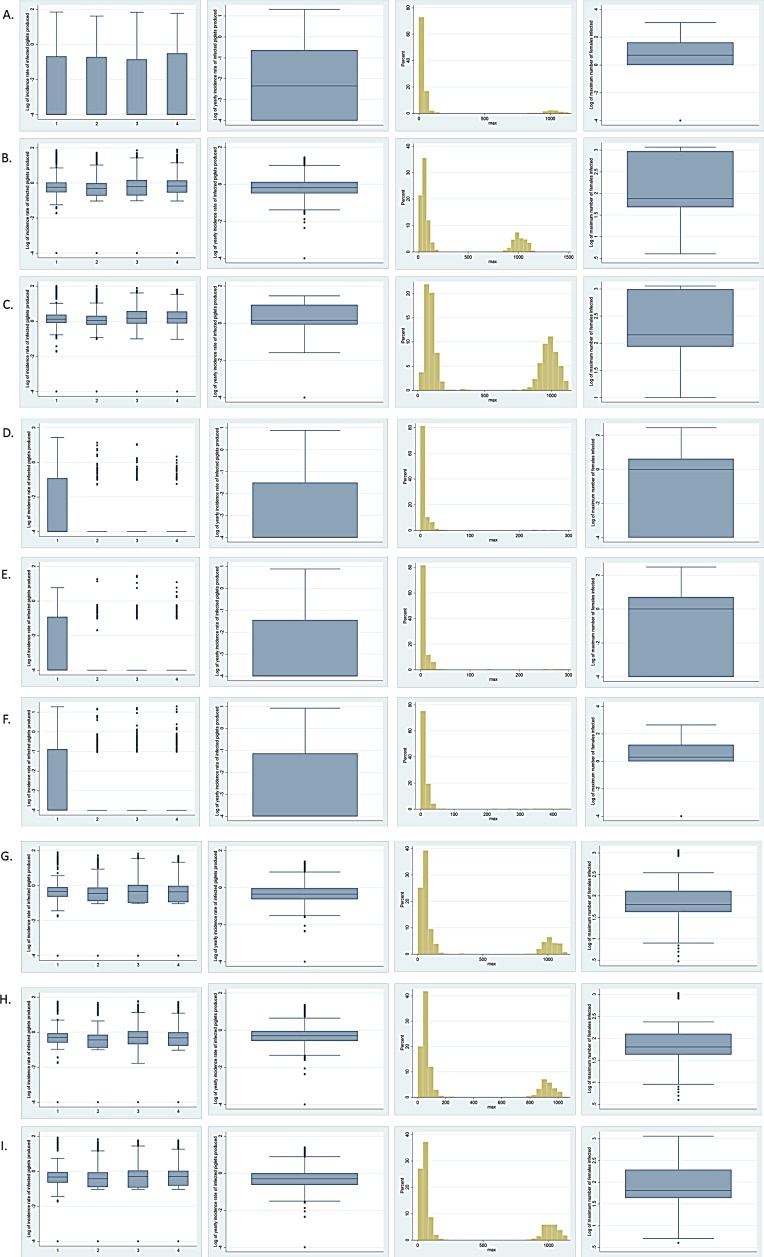
Boxplot graphs and histograms showing model piglet-level outcomes for different PRRSV control scenarios. **Left to right: Log of the incidence of infected weaned piglets for four cycles, with each cycle representing 3 months and log of yearly incidence of infected weaned piglets.** A. Baseline 1; B. Baseline 5; C. Baseline 10; D. Live-virus inoculation, 30d acclimation; E. Live-virus inoculation, 60d acclimation; F. Live-virus inoculation, 60d acclimation, 56d for duration of infectiousness; G. Modified-live vaccine scenario, 95% immunization efficacy and 60 days of duration of acclimation; H. Modified-live vaccine scenario, 95% immunization efficacy and 30 days of duration of acclimation; I. Modified-live vaccine scenario, 70% immunization efficacy and 60 days of duration of acclimation.

For all baseline scenarios, the median yearly incidence of PRRS positive weaned pigs was relatively small: 0.004%, 0.63% and 1.44% when one, five and ten infected females were introduced into the herd (Table 4, Fig 4A–4C). Maximum yearly incidences reached 20.44%, 27.70% and 29.76% for the scenarios where one, five and ten infected females were introduced into the herd (Table 4).

**Table 4:** Median (inter-quartile range), maximum and minimum incidence of infected piglets, and average total number of piglets produced, considering a one-year period.

Scenario^a^	Median incidence (IQR)	Maximum incidence	Minimum incidence	Mean total number of piglets produced
Baseline_1	0.004% (0.23)	20.44%	0%	23,236
Baseline_5	0.63% (0.96)	27.70%	0%	22,946
Baseline_10	1.44% (8.52)	29.76%	0%	22,585
LVI^b^_30_95	0% (0.03)	7.37%	0%	22,438
LVI^b^_60_95_a	0% (0.03)	7.58%	0%	22,231
LVI^b^_60_95_b	0% (0.07)	8.31%	0%	22,757
MLV_60_95	0.45% (0.69)	25.79%	0%	23,050
MLV_60_80	0.47% (0.74)	23.83%	0%	22,961
MLV_60_70	0.49% (0.72)	24.65%	0%	22,969
MLV_30_95	0.50% (0.61)	22.85%	0%	23,120

^a^Considered 1,000 simulations for each scenario. For scenario definitions please refer to Table 2.

^b^For LVI scenario, piglets were counted as infected if they were infected by the challenge virus only, therefore this measure might be underestimated.

Interestingly, when introducing one infected female into the herd, only a small number of the simulations resulted in more than 100 infected females (9.20%), but for a considerable percentage of those simulations that resulted in more than 100 infected females (27/92, 29.35%), the outbreak only occurred after one full year after virus introduction. This was also true for the scenarios where five or ten animals were introduced, even though the percentage of simulations where more than 100 animals were infected was considerably higher (35.4% for the 5-infected animal scenario, for which 30.50% reached the maximum after one year of virus introduction and 65.8% for the 10-infected animal scenario, for which 22.04% reached the maximum after one year after virus introduction). Due to the fact that introducing a total of five infected animals produced a reasonable number of simulations where more than 10% of the population became infected in the baseline scenario, this number of infected animals was chosen to evaluate the MLV and LVI control strategies. Additional sensitivity analysis suggested that cumulative incidence of infection was sensitive with respect to probability of infection, and between-room probability of infection, whereas it was not sensitive on the assumed number of contacts (data not shown).

### MLV scenario

For the MLV scenario, as vaccine efficacy increased, the number of outbreaks occurring in the herd following virus introduction decreased (Table 3, Fig 3G–3I). The increase in the percentage of simulations resulting in outbreaks was especially evident when vaccination efficacy dropped from 80 to 70%. As duration of acclimation was shortened from 60 to 30 days, the overall number of outbreaks increased, and they tended to occur earlier after virus introduction (Fig 3G and 3J). This change, however, was not dramatic.

Interestingly, despite the changes described above, the average maximum number of females infected remained relatively similar amongst scenarios with different immunization efficacies: 268.03 animals (SD: 399.12) for 95% efficacy, 279.99 animals (SD: 406.09) for 80% efficacy, and 295.32 animals (SD: 418.51) for 70% efficacy. Using duration of acclimation of 30 days, the mean maximum number of females infected was 255.22 animals (SD: 365.72).

The median yearly incidence of infected weaned piglets was 0.45% (maximum 25.79%) for the 95% efficacy scenario, 0.47% (maximum 23.83%) for the 80% efficacy, and 0.49% (maximum 24.65%) for the 70% efficacy. For the scenario with 30 days of acclimation, the median yearly incidence of infected weaned piglets was 0.50% (maximum 22.85%; Table 4). The data were highly skewed (Fig 4G–4J).

Finally, simulations for all MLV scenarios showed that many outbreaks occurred after one year of initial virus introduction, with approximately 30% of simulations resulting in more than 100 animals becoming infected for all evaluated scenarios.

### LVI scenario

There were no simulations that resulted in the majority of the herd becoming infected for the live-virus exposure scenario. For scenarios using acclimation durations of 30 and 60 days, the maximum number of female pigs infected was never more than 300, and only 6% and 5% of the simulations resulted in more than 100 animals infected for each scenario, respectively (Table 3). However, as pointed out in the methodology section, it is important to note that herds using such control measure have an underlying population of virus-exposed animals that are possibly acting as carriers of the resident virus used for LVI during the acclimation stage. These animals were not captured as infectious in the statistical analysis. For the scenario where the young female pigs were being moved to the main herd at 30 days, there appeared to be more outbreaks in the early phase after virus introduction compared to the scenario where those animals were being moved after 60 days of acclimation (Fig 3G and 3J). As duration of infectiousness was fixed at 56 days, however, the percentage of simulations resulting in outbreaks increased, even though those outbreaks were not too large (maximum 500 females infected).

The median yearly incidence of infected weaned piglets was around 0.50% for all vaccination scenario simulations (Table 4). The maximum incidence was approximately between 22 and 25% (Table 4).

## Discussion

The discrete event agent-based model developed herein allows for evaluation of different PRRS control strategies in farrow-to-wean swine facilities. The main question addressed herein for both LVI and MLV scenarios was the frequency by which a re-introduction of PRRS virus would result in infection of a large number of animals in swine herds that are considered “immunologically stable” (i.e. have some kind of immunity in place).

The model was not only able to mimic pig flow and animal replacement as seen in breeding herds under field conditions, but also to allow for the incorporation of stochasticity for PRRS parameters that are subject to variability and uncertainty, such as duration of infectiousness. The current model could be modified as necessary, and scenarios for specific conditions and interventions could be created, for example with changes in herd size, contact patterns, disease, and production parameters. The model also allows for evaluation of different control scenarios, such as multiple vaccination schedules, which were not explored in the current project.

An interesting finding from the current model was that outbreaks can occur after long periods of initial virus introduction into the herd. This has been previously reported [17], and is a reasonable explanation for situations where high health swine herds have PRRS outbreaks after long periods of presumably successful virus elimination programs and monitoring, which intrigue field veterinarians. The authors hypothesize that such phenomena might not necessarily be due to breaks in internal site biosecurity, but simply due to the existence of animals that are able to act as virus reservoirs for long periods of time. Furthermore, this finding raises important points, such as the importance of herd monitoring and use of sensitive and risk-based sampling methodologies when the goal is to detect low-prevalence of PRRSV in a herd that has invested time and resources in virus elimination. This observation could also have important implications for outbreak investigation regarding PRRS transmission between farms. Typically, during disease investigation, a period of tracing back contacts for the case farm is measured over several weeks. Nonetheless, such finding could mean that the duration of such period should extend, perhaps more in line with what has been reported during PRRS incursion in Sweden [18]. However, such investigations would require extensive resources and the decision should be carefully considered. Perhaps in areas where PRRS is endemic and resources are limited, such extension of the contact tracing period should be conducted for cases where PRRSV introduction is difficult to trace. Elucidating origin and consequences of outbreaks would aid in the implementation of strategies that would mitigate between-herd PRRS spread.

Another important observation from the results of the model is that when small loads of virus are introduced to the herd, as exemplified by the introduction of one infected animal, outbreaks occur with lower frequency. This observation reinforces the idea that internal and external biosecurity, and regular and adequate testing of replacement animals are valuable, especially when other factors that may alter PRRSV spread remain out of the producers or veterinarian’s control (e.g. weather conditions, pig flow, and area pig density).

A somewhat unexpected observation for the LVI scenario was that accounting for variation in duration of infectiousness appeared to be more conservative compared to using a fixed duration. This could be attributed to the stochastic nature of the process, and to the fact that using the distribution allows for animals with short duration of shedding, which contributes to faster extinction of a potential epidemic considering the population consists of a number of animals that are partially or completely immune and the relatively low number of potentially infectious individuals might not be enough to cause a major epidemic.

The current model has limitations that also should be acknowledged. Firstly, the model does not include indirect introduction of PRRSV, for example via fomites. The authors argue that such introduction would be more short-lived than the introduction of an infected animal, and therefore the number of outbreaks produced would probably be reduced significantly compared to the introduction of infected live animals. Another limitation of this study is that different PRRSV strains were not considered, even though the authors recognize that there are indications that virus strain can play an important role in the minimum amount of infectious dose, and likely the probability of transmission [15]. Furthermore, statements with regard to clinical disease cannot be made from the current model, since infection was modeled. The dynamics of infection are important for propagation of the microbe and for herd health, while clinical disease is mostly important from an economic and animal welfare standpoint. However, clinical manifestation of disease could be incorporated into the present model and could provide insights on economic losses for producers. Finally, parameters governing transmission within and between populations were primarily extracted from the available literature and expert opinion, and this are needs further improvements. This could explain the sharp spikes seen in the epidemic curves. The approach used herein was to use data available from empirical studies to create a model, test strategies, and aid in the development of new hypothesis. The sensitivity analysis conducted for the baseline model suggested that cumulative incidence was sensitive on the probability of infection, and particularly on the probability of transmission between pigs located in different rooms. Estimation of between room probability of transmission should be one of the areas for further research for this type of modelling. In this study, we opted to use previously used values. However, it is possible that such probability is higher in an average herd. If this is the case, then the results of the current study could be extrapolated to herds with excellent internal biosecurity protocols.

It is important to note that the MLV and LVI scenarios are structurally different models; therefore direct comparison of outcomes should be made with caution. Also, both intervention scenarios considered that the population was managed as an open population throughout the study period, with no further control strategies (e.g. herd closure for a determined period of time) that may come as a result of a clinical outbreak. Even though the LVI scenario seemed to produce promising results with the lowest percentage of simulations resulting in outbreaks being produced, the reader should consider that, under such a control strategy, swine producers would always have to deal with having an underlying infected population (“exposed to the virus”) in the herd. This could have an impact on piglet production [3], besides increasing the risk of “virus leakage” to growing pig populations on or off-site. Both scenarios could damage the swine producer financially, but such quantification was not conducted in the current research. Furthermore, there was an underlying assumption that the LVI was conducted using a homologous strain to the one used for the challenge, which may not be the case in field conditions. This might had overestimated the benefit of this strategy.

The MLV scenario showed that vaccination efficacy rates have an impact in the likelihood of outbreaks occurring in swine herds, with this being more evident as vaccine efficacy decreased to 70%; therefore, ensuring high coverage, correct vaccine administration and adequate vaccine storages are important recommendations for producers that decide to use such control strategy.

Surprisingly, the incidence of PRRS positive piglets produced for all scenarios examined herein was, on average, relatively small. The authors believe this may be explained by three main reasons. Firstly, it could be a result of the assumptions for the immunological states for piglets, such as the assumption that infected female pigs give birth to half the number of piglets compared to non-infected females. Secondly, due to the dynamic nature of the process, it could be that the virus is actually getting to the farrowing rooms (where piglets are) after a longer period of virus introduction, as opposed to early on. Lastly, the measure captured herein refers to an incidence measure, in contrast with most estimates obtained (during the peak of infection) under field conditions, which commonly refer to prevalence and are usually higher. Even though the incidence of PRRS positive piglets was relatively small, under the assumptions of this model, not a single strategy guaranteed absence of piglet infection. This finding supports piglet-level control strategies such as the Management Changes to Reduce Exposure to Bacteria and Eliminate Losses (McRebel^TM^), [19]. The impact of this and other strategies, however, were not evaluated in the current model.

## Conclusions

In conclusion, the model developed herein showed that both immunization strategies (LVI and MLV), with different success, would decrease the likelihood of outbreaks in a farrow-to-wean swine herd. However, none would be successful in completely ceasing virus circulation in a herd that is managed as an open herd with no further intervention strategies. This supports the role of additional infection control methods in the farrowing rooms, such as McRebel^TM^ and effective surveillance to detect prevalence at potentially low levels in herds that implement such immunization strategies.
